# Novel *STAT3* variant causing infantile-onset autoimmune disease

**DOI:** 10.3389/fmed.2023.1251088

**Published:** 2023-11-09

**Authors:** Miao Pan, Justin Kurtz

**Affiliations:** ^1^Division of Pathology and Laboratory Medicine, Children’s National Hospital, George Washington University, Washington, DC, United States; ^2^Department of Pathology, The George Washington University School of Medicine and Health Sciences, Washington, DC, United States; ^3^Department of Pediatrics, The George Washington University School of Medicine and Health Sciences, Washington, DC, United States

**Keywords:** *STAT3*, SH2, infantile-onset, autoimmune, sequencing

## Abstract

Signal transducer and activator of transcription 3 (*STAT3*) is a member of the STAT protein family implicated in the development of infantile-onset multisystem autoimmune disease. *STAT3*-related autoimmune disease is characterized by multiorgan autoimmunity, lymphoproliferative disease, and recurrent infections. The presentation is variable, with some patients also developing neonatal diabetes mellitus and interstitial lung disease. Gain-of-function variants in the Src homology 2 domain, leading to autophosphorylation and activation of *STAT3*, have been previously reported in patients with disease. Here, we report a patient with a novel missense variant, p.Glu616Ala, in *STAT3* presenting with infantile-onset multisystem autoimmune disease.

## 1. Introduction

The signal transducer and activator of transcription 3 (*STAT3*) gene encodes for a member of the STAT protein family. It is rapidly activated via phosphorylation by Janus kinase (JAK) in response to various cytokines, including interleukin-6, leukemia inhibitory factor, oncostatin M, and interleukin-11 (1). Functional domains in *STAT3* include an N-terminal coiled-coil domain (codons 130–320) for protein–protein interaction, a DNA binding domain (codons 320–490), a Src homology 2 (SH2) domain (codons 580–680), a tyrosine phosphorylation site (codon 705), and a carboxyl transactivation domain with a serine phosphorylation site (codon 727) (2). Phosphorylation at Tyr705 leads to phosphotyrosine interactions between the SH2 domains of monomers resulting in dimerization of the *STAT3* protein, which facilitates DNA binding (2). *STAT3* dimers translocate to the nucleus and function as transcription factors, mediating changes in gene expression in response to cytokines (3). *STAT3* is also involved in signal transduction cascades activated by intracellular proteins, such as RAS and SRC (4).

Germline variants in *STAT3* have been reported to cause two different types of immune-related disorders. Hyper-immunoglobulin (Ig) E recurrent infection syndrome (MIM:147060) is characterized by high elevations of serum IgE, recurrent staphylococcal infections, and an inappropriately weak immune response (5). *STAT3*-associated infantile-onset multisystem autoimmune disease (MIM: 615952), on the other hand, can present with multiorgan autoimmunity, lymphoproliferative disease, and recurrent infections (6). While the inheritance of *STAT3* disease is considered autosomal dominant, the vast majority of patients with *STAT3*-related disease are born to unaffected parents and are found to have a *de novo* alteration (5). *STAT3*-related disease is observed with both loss-of-function (dominant negative) and gain-of-function (constitutive activation) variants. The majority of dominant negative disease-causing variants are located in the DNA binding domain of the *STAT3* protein, which results in classical multisystem hyper-IgE syndrome (HIES) (5). Activating variants in *STAT3* result in a different constellation of symptoms compared to dominant negative disease, characterized by early-onset multiorgan autoimmunity, recurrent infections, short stature, and lymphoproliferative disease (6). Approximately 20% of patients develop early onset, monogenic type 1 diabetes, which is rarely observed in other forms of autoimmune primary immunodeficiencies (7). Germline activating variants have been reported in all functional domains of the *STAT3* protein (7). Interestingly, activating somatic alterations identified in lymphoproliferative disorders are predominantly point mutations in the SH2 domain (8). Similar to most other autoimmune disorders, diagnosing *STAT3*-related disorders can be challenging due to the complexity of these conditions, the diversity of clinical presentations, and the lack of a single diagnostic test. Therefore, integration of new clinical tests holds great potential for diagnosing the diseases and identifying novel clinical presentations and biomarkers.

## 2. Case description

The patient is a full-term female born by cesarian section and found shortly after birth to have protein calorie malnutrition, pancreatic insufficiency, hypotonia, intermittent hypoglycemia, and hepatosplenomegaly with transaminitis. At birth, her weight was in the 99th percentile and steadily declined to the 75th percentile at 2 months of age and below the 5th percentile by 5 months of age. The growth charts followed similar trends for height and head circumference. Around 6 months of age, she was admitted to the hospital for hypoxic respiratory failure requiring bilevel positive airway pressure (BiPAP) secondary to rhino-enterovirus infection. During the admission, she was found to have elevated pancreatic elastase and fecal fat, and the hospital stay was complicated by intermittent hypoglycemia and poor weight gain. She was admitted to our facility for failure to thrive as well as non-bloody, non-bilious vomiting, a sunken fontanelle, and a distended abdomen. She was found to have anemia (negative direct antiglobulin testing), thrombocytopenia, and transaminitis. She had an extensive GI workup due to persistent loose stools since birth, which found no evidence of autoimmune hepatitis (negative anti-actin and anti LKM antibodies), inflammatory bowel disease, or Hirschsprung disease. Antibody testing identified elevations in two antibodies related to autoimmune disease, GAD65 (67 IU/mL) and insulin (>50 units/mL). Treatment with corticosteroids resulted in improvement of her cytopenias, gastrointestinal symptoms, and transaminitis. However, while on corticosteroids she developed significant hyperglycemia requiring insulin. A liver biopsy demonstrated increased CD8+ T cells (more than CD4+), compatible with the history of immunodysregulation but not specific for a lymphoproliferative disorder. At this point, personalized genetic testing panel containing 211 genes was ordered to assist in elucidating the cause of her symptoms, and she was found to have a variant in *STAT3*, consistent with a diagnosis of multisystem autoimmune disease of infantile onset. She was started on tofacitinib with improvement in cytopenias and enteropathy symptoms.

## 3. Materials and methods

Genomic DNA was extracted from the patient blood and processed using the PCR-Free whole genome library preparation kit to generate the whole genome. These regions were sequenced by massive parallel (NextGen) sequencing on the Illumina NovaSeq 6,000. DNA sequencing reads were aligned to human genome build UCSC hg19 (hs37d5) in BaseSpace Sequence Hub using DRAGEN Germline Pipeline (v3.4.5). Sequencing data quality was assessed for coverage of 30X and filtered based on curated reference sequence (RefSeq) transcripts. Identified variants are analyzed by population databases, *in silico* prediction algorithms, Human Gene Mutation Database (HGMD) Professional, and the data aggregator Alamut Visual v2.11.

## 4. Results

A heterozygous missense variant NM_139276.2:c.1847A > C (p.Glu616Ala) was identified in *STAT3* gene. *STAT3* functions not only as a transcription factor but also a signaling molecule mediating maturation of immune system cells. *STAT3* p.Glu616 is located in Src Homology 2 (SH2) domain, which is essential for protein–protein interaction and control STAT3 oligomeric state. The variants upstream and downstream within 2 amino acids (p.S614G, p.K615E, p.G618A, p.G618D and p.G618V) have been reported to be associated with either *STAT3* deficiency or Hyper-IgE syndrome. Interestingly, this variant has never been observe in general population; however, four other missense variants at the same location (p.Glu616Val, p.Glu616Gly, p.Glu616Lys, and p.Glu616Gln) have been reported in patients with fatal interstitial lung disease and multisystem autoimmunity disorder ((8, 9) and ClinVar ID 1328394 and 421084) (see Supplementary Table S1). *In vitro* functional study suggest that p.Glu616Val increases baseline transcriptional activity and maintains longer phosphorylation after cytokine stimulation. This indicates that the change of Glu616 residue may have a gain-of-function effect on *STAT3*. Furthermore, parental testing confirmed this variant is *de novo* in this patient. Based on this information, *STAT3* p.Glu616Ala is classified as a Likely Pathogenic variant according to the guidelines recommended by the American College of Medical Genetics and Genomics (see Supplementary Figure S1).

## 5. Discussion

Loss-of-function and gain-of-function variants in *STAT3* lead to different immune disorders. Heterozygous gain-of-function variants in *STAT3* have been associated with multisystem infantile-onset autoimmune disease, while heterozygous loss-of-function variants in *STAT3* are associated hyper-IgE recurrent infection syndrome (10). The two disorders also have different clinical presentations. *STAT3*-related autoimmune disease is characterized by a spectrum of autoimmune disorders affecting multiple organs. Common manifestations include diabetes mellitus, autoimmune enteropathy or celiac disease, and autoimmune hematologic disorders (3, 11). Hyper-IgE recurrent infection syndrome is characterized by frequent infections, and many people with this condition also have distinctive coarse facial appearance, abnormal dentition, hyperextensibility of the joints, and bone fractures (12).

Different missense variants at the same amino acid position in the SH2 domain of *STAT3*, p.Glu616Gly and p.Glu616Lys, have been functionally characterized. Song et al. demonstrated that both p.Glu616Gly and p.Glu616Lys result in increased autophosphorylation of the tyrosine residue at codon 705 of the *STAT3* protein (8, 13). These missense variants were shown to upregulate expression of downstream targets of STAT3, including MYC, BIRC5, and HIF1A (8). In addition, cell lines harboring vectors with p.Glu616Lys mutant *STAT3* demonstrated IL-3 independent growth (8). Activating variants in the SH2 domain occur at the dimerization interface and are thought to result in increased hydrophobicity, which leads to increased autophosphorylation (14).

The patient reported here has both the general symptoms identified in multisystem autoimmune disease as well as findings that are more specific for gain-of-function variants in *STAT3*. Many of these patients develop a neonatal form of diabetes mellitus in the first 6 months of life due to the deleterious effect that *STAT3* autoactivation confers on insulin secreting cells of the pancreas (15). In addition, the patient discussed here was found to have elevated antibodies for GAD65 and insulin, which are known markers for type 1 diabetes and latent autoimmune diabetes in adults (16). *STAT3* promotes proliferation and survival of CD8+ T cells, as seen on the liver biopsy in our patient (17). The dysregulation of the CD8+ T cells plays a role in the accelerated onset of diabetes (18). Furthermore, these patients commonly suffer from interstitial lung disease (9), while our patient required BiPAP at 6 months of age for hypoxic respiratory failure. Gain-of-function variants in *STAT3* result in activation of the JAK–STAT pathway, and therefore, JAK inhibitors may be considered as a targeted systemic therapy in these patients (13, 19). Our patient received treatment with a JAK inhibitor (tofacitinib), and after 6 months of treatment, she has shown sustained improvement in her glucose levels, pancytopenia, and enteropathy symptoms.

*STAT3*-associated autoimmune disease displays varying phenotypic presentations, even in patients with mutations in the same functional domain (3). With the variable expressivity and incomplete penetrance seen with *STAT3* disease, it is difficult to predict what the presentation and prognossis will be for a novel variant. Given the features of general immune dysregulation combined with our patient’s history of diabetes mellitus, it is speculated that p.Glu616Ala confers a gain-of-function on STAT3 through increased autophosphorylation. In conclusion, *STAT3* p.Glu616Ala is a novel mutation resulting in multisystem infantile-onset autoimmune disease in the patient presented here.

## Ethics statement

Ethical review and approval was not required for the study on human participants in accordance with the local legislation and institutional requirements. Written informed consent from the participants’ legal guardian was not required to participate in this study in accordance with the national legislation and the institutional requirements. Written informed consent was obtained from the individual’s legal guardian for the publication of the data included in this manuscript.

## Author contributions

All authors listed have made a substantial, direct, and intellectual contribution to the work and approved it for publication.
