# Supplementary material for: Novel STAT3 variant causing infantile-onset autoimmune disease
Source: Front Med (Lausanne). 2023 Nov 9;10:1251088. doi: 10.3389/fmed.2023.1251088 (PMC10666157; doi:10.3389/fmed.2023.1251088)
Supplement: Supplementary file 1 [file Table_1.docx]

# Supplementary Table 1. Comparison of multiple missense variants in STAT3 p.Glu616 variants

| Variant | p.Glu616Ala | p. Glu616Val | p.Glu616Gly | p.Glu616Lys | p.Glu616Gln |
| --- | --- | --- | --- | --- | --- |
| c. change | c.1847A>C | c.1847A>T | c.1847A>G | c.G1846A | c.1846G>C |
| Inheritance | De novo | NA | De novo | NA | NA |
| Age of onset | Neonatal | 2 years old | NA | NA | NA |
| Clinical presentation | pancreatic insufficiency, hypoglycemia, multisystem autoimmune disease, hepatosplenomegaly, anemia, thrombocytopenia, hypotonia, feeding intolerance, chronic diarrhea | fatal interstitial lung disease, dry cough, wheezing, exercise intolerance, atopic eczema | NK/T-cell lymphoma | NK/T-cell lymphoma | NA |
| Proposed disease mechanism | Gain of function | Gain of function | Gain of function | Gain of function | NA |
| ClinVar classification | NA | NA | Uncertain significance; 1328394 ​ | NA | Likely pathogenic; 421084 ​ |
| Reference | This study | PMID: 34549903 | PMID: 30054295, 36228738 and ClinVar | PMID: 30054295 | ClinVar |
