# Supplementary material for: Novel STAT3 variant causing infantile-onset autoimmune disease
Source: Front Med (Lausanne). 2023 Nov 9;10:1251088. doi: 10.3389/fmed.2023.1251088 (PMC10666157; doi:10.3389/fmed.2023.1251088)
Supplement: Supplementary file 2 [file Table_2.docx]

Supplementary Table 2. Clinical molecular genetic testing performed prior to this study

| **Differential Diagnosis** | **Testing results** |
| --- | --- |
| glycogen storage diseases | Commercial comprehensive glycogen storage disorder panel: inconclusive |
| fatty acid oxidation defects | Commercial fatty acid oxidation disorder panel: negative; |
| Fragile X | CGG repeat expansion: negative |
| Prader-Willi/Angelman syndrome | Commercial deletion/duplication/methylation panel: negative |
| Russell-Silver syndrome | Commercial deletion/duplication/methylation panel: negative |
| Lysosomal and peroxisomal storage disorders | Commercial sequencing panel: negative |
