# Supplementary material for: Novel STAT3 variant causing infantile-onset autoimmune disease
Source: Front Med (Lausanne). 2023 Nov 9;10:1251088. doi: 10.3389/fmed.2023.1251088 (PMC10666157; doi:10.3389/fmed.2023.1251088)
Supplement: Supplementary file 3 [file Table_3.docx]

Supplementary Table 3. Other clinical non-molecular genetic testing performed prior to sequencing

| **Differential Diagnosis** | **Testing results** |
| --- | --- |
| Alpha-1 antitrypsin deficiency | Negative |
| Congenital disorder of glycosylation | Carbohydrate deficient transferrin: negative |
| Cystic fibrosis | Sweat chloride test: negative |
| Very long chain fatty acids profile | Negative |
| Plasma amino acids | Normal |
| Total and free carnitine | Normal |
| Acylcarnitine | Normal |
| Urine mucopolysaccharides | Negative |
| Urine organic acids | Ketonuria and dicarboxylic aciduria |
| Fecal fat qualitative | Abnormal, fecal elastase low |
| Fecal pH | Normal and fecal reducing substances negative |
